# Measuring *Caenorhabditis elegans* Life Span on Solid Media

**DOI:** 10.3791/1152

**Published:** 2009-05-12

**Authors:** George L. Sutphin, Matt Kaeberlein

**Affiliations:** Department of Pathology, University of Washington; Molecular and Cellular Biology Program, University of Washington

## Abstract

Aging is a degenerative process characterized by a progressive deterioration of cellular components and organelles resulting in mortality. The nematode *Caenorhabditis elegans* has emerged as a principal model used to study the biology of aging. Because virtually every biological subsystem undergoes functional decline with increasing age, life span is the primary endpoint of interest when considering total rate of aging. In nematodes, life span is typically defined as the number of days an animal remains responsive to external stimuli. Nematodes can be propagated either in liquid media or on solid media in plates, and techniques have been developed for measuring life span under both conditions. Here we present a generalized protocol for measuring life span of nematodes maintained on solid nematode growth media and fed a diet of UV-killed bacteria. These procedures can easily be adapted to assay life span under various common conditions, including a diet consisting of live bacteria, dietary restriction, and RNA interference.

**Figure:**
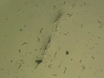


## Protocol

### Part 1: Prepare nematode growth media (NGM) plates

This section describes the preparation of the solid NGM plates for use in the life span experiment. A basic life span experiment requires two types of plates: standard NGM plates, which contain no additives, and Amp/FUDR plates, which have both ampicillin (Amp) and fluorodeoxyuridine (FUDR) added to the NGM. Ampicillin is used to prevent foreign bacterial contamination. FUDR inhibits cell division, reduces egg production, and prevents eggs from hatching. The use of FUDR for longevity analysis does not affect adult life span and removes the need to transfer worms every few days in order to separate them from growing larva.  Both types of plates are seeded with *E. coli *OP50 bacteria, which is subsequently killed by exposure to UV.

Prepare NGM (see Section 5 for recipe and storage notes) and label petri plates. You will need 1 60 mm petri plate per 10 mL of NGM. If you are starting with previously prepared solid NGM, continue to step 1.2. If you are starting with freshly autoclaved NGM, skip to step 1.4.
Completely melt solid NGM by microwaving on high in 30 second pulses. Swirl media between pulses to prevent pressurized gas bubbles from building up.
Place liquid NGM in 55 °C water bath or on the bench to cool.
Once NGM reaches 55 °C add 33 µL of 150 mM FUDR and 100 µL of 100 mg/mL Ampicillin per 100 mL NGM (for Amp/FUDR plates) and swirl to mix. For NGM plates without additional drugs proceed directly to step 1.5.
Using sterile technique, pipette 10 mL NGM into each 60 mm petri plate. Avoid forming bubbles if possible. If bubbles do form, they can be popped using a pipette tip.
Leave plates on the bench with lids on to allow NGM to solidify and dry. If possible, leave plates on bench for 2 days before adding bacteria, but 1 day will work if plates are needed sooner.
On the day before the plates finish drying, seed liquid LB culture with a single colony from a fresh streak of *E. coli *OP50 bacteria on LB agar. You should prepare at least 1 mL of an overnight OP50 culture per 60 mm plate.
Place OP50 culture in 37 °C shaker and allow bacteria to grow overnight (culture should reach saturation).
Pellet OP50 bacteria by spinning at 3,500 g for 10 minutes.
Remove 90% of supernatant and resuspend bacteria to concentrate culture 10x.
Pipette 100 µL of 10x concentrated OP50 culture into the center of solidified NGM plates. Swirl plates gently to spread out bacteria culture if needed (ideally bacteria should cover the central area of the NGM without coming near the plate walls). Try to avoid touching the pipette tip to the surface of the NGM, as flaws in the surface of the media allow the worms to burrow into the NGM.
Leave plates on the bench overnight to allow bacteria culture to dry on the solid NGM (typically takes about 24 hours).
Once dry, expose the surface of the plates to a UV dose sufficient to arrest growth of the bacteria. If you are using a Stratagene UV Stratalinker 2400:
  Place plates in the Stratalinker and remove lids
  Close the door and turn on the Stratalinker
  Press ‘Energy’
  Enter ‘9999’ using the keypad
  Press ‘Start’
  The plates will be exposed to UV for approximately 5 minutes
Plates with UV-killed bacteria can be stored upside down at 4 °C for up to 1 month.

### Part 2: Perform a timed egg laying to acquire an age-synchronized population of animals

In this section we generate a population of worms with a common hatch-date. This is accomplished by allowing reproductively active adults to lay eggs on a plate for a defined period of time and allowing those eggs to develop.

(Optional) Transfer approximately 20 young adult worms to a fresh NGM plate without FUDR. Leave plates at 25 °C in order to allow worms to propagate and eat through all of the bacterial food. After the bacterial food has been consumed newly hatched worms will enter the growth arrested dauer stage. You will start to see dauer larva around within a week, depending on how many worms you transfer to the plate initially. Dauer larva can be used for the next steps for up to 1 month.
(Optional) Transfer 20 to 30 dauer larva to a fresh NGM plate without FUDR. In the presence of food dauer larva will become reproductively active adults within 2 days and remain reproductively active for a few days thereafter at 25 °C.
Transfer 10 to 15 reproductively active adults to a fresh NGM plate without FUDR. This plate is referred to as the timed egg laying (TEL) plate.
Leave the TEL plate at 25 °C for 6 hours in order to allow the worms time to lay eggs.
Remove adult worms from TEL plate. Plate can be visually inspected for eggs before removing adults. If the worms have not laid a sufficient number of eggs the TEL plate can be left at 25 °C for up to a total of 24 hours before removing adult worms.
Place TEL plate at 20 °C until the eggs have hatched and the worms have developed to the L4 larval stage (this usually takes 2 days for wild type C. elegans, but can take longer for strains with slow development phenotypes).

### Part 3: Score animals for life span

In this section we follow the age-synchronized population of worms from Part 2 until they die. Worms are maintained on Amp/FUDR plates to prevent egg production and bacterial contamination and are considered dead when they fail to respond to external stimuli.

Transfer L4 larva to seeded Amp/FUDR plates. For each strain or condition being tested, it is typical to set up 2-3 plates with 25-30 worms per plate. Images of the *C. elegans* life stages are provided for reference (Figure 1).^1^
Place Amp/FUDR plates at 20 °C for 24 hours.
After 24 hours, visually assess worms, media, and bacteria. Transfer worms to fresh Amp/FUDR plates if any of the following is observed:
  Worms have eaten most of the bacterial food. Early in the life span experiment worms will have to be transferred 1 to 2 times a week to prevent the bacteria from being depleted.
  A significant number of larvae are present. This is usually an indication that the worms were transferred to the Amp/FUDR plates as young adults instead of L4s and were able to lay some eggs before the FUDR took effect. The FUDR will generally prevent the larvae from growing into full adults, but occasionally a few will grow into adults and become confused with the experimental animals.
  Live /growing bacteria are observed.  Generally, the combination of Amp and UV-killing will ensure that no live bacteria contaminate the experiment, but occasionally it occurs.
  Fungal growth is observed on the media. If caught early enough, fungal growth can usually be cut out using a pipette tip or spatula. Once it grows to be larger than a few millimeters in diameter it is usually easier to transfer the worms to a new plate.
  Using the dissection scope, determine whether each worm is alive or dead.
  Gently tap the plate. The worm is alive if it moves in response to the tapping.
  If the worm does not respond to tapping the plate, zoom in on the head region.
  Gently tap the worm’s head with platinum transfer pick. Score the worm as dead if it does not respond by moving its head.
  Dead worms can be removed from the plate.
Record the date and the number of worms that are alive and dead.
Return plates to 20 °C.
Repeat steps 3.3 through 3.6 every 2 to 3 days until all worms have died.

### Part 4: Representative Results.

The raw data produced by a nematode life span experiment is a list of dates with corresponding numbers of worms that are alive and dead for each strain tested. The number of worms that die on each day is typically inverted to calculate the proportion of worms alive on each day (Figure 2A), which is plotted graphically as a survival curve (Figure 2B; the day of the timed egg laying is considered day 0). The life span for each individual worm in the study can be calculated from the count of worms that die on each day and used to calculate mean and median life span for comparison between strains. The count of number of worms alive on each day is not used directly in life span analysis. Worms maintained on solid media will occasionally ‘flee’, or crawl either up the wall of the plate or down beneath the media. The number of worms alive on each day can be used to determine how many worms fled throughout the course of the experiment. Worms that flee are typically removed from analysis. As a benchmark, the typical median life span for N2, the *C. elegans* wild-type strain, maintained on UV-killed bacteria at 20 °C is approximately 25 days as measured from egg.

### Part 5: Solutions

**Table:** 

**Nematode Growth Media (NGM), 100 mL:**
Combine:
0.3 g	NaCl
0.25 g	Peptone
2 g	Agar
**Autoclave for 40 minutes and let cool to 55 °C, then add:**
100 µL	1 M MgSO_4_
100 µL	5 mg/mL Cholesterol
100 µL	1 M CaCl_2_
1.625 mL	1.5 M KPi pH 6.0
Liquid NGM can be used immediately to pour plates or allowed to solidify and stored long-term at room temperature

**Table:** 

**Luria Broth (LB), 1L:**
10 g	BactoTryptone
5 g	Yeast Extract
10 g	NaCl
10 mL	1 M Tris pH 8.0
1 L	deionized water
Autoclave and store at room temperature.

**Table:** 

**1 M MgSO_4_, 300 mL:**
73.95 g	MgSO_4_
300 mL	deionized water
Autoclave and store at room temperature.

**Table:** 

**5 mg/mL Cholesterol, 200 mL:**
1 g	cholesterol
200 mL	100% ethanol
Filter sterilize and store at room temperature.

**Table:** 

**1 M CaCl_2_, 500 mL:**
27.75 g	CaCl_2_
500 mL	deionized water
Filter sterilize and store at room temperature.

**Table:** 

**1.5 M KPi pH 6.0, 1L:**
Combine:
31.4 g	KPi dibasic
179.6 g	KPi monobasic
850 mL	deionized water
Heat while mixing to allow KPi to dissolve into solution. Adjust pH to 6.0 with 10 N NaOH.
Add deionized water to 1 L.
Autoclave and store at room temperature.

**Table:** 

**1 M Tris, pH 8.0:**
60.57 g	Tris
400 mL	deionized water
Adjust pH to 8.0 with HCl.
Add deionized water to 500 mL.
Filter sterilize and store at room temperature.

**Table:** 

**150 mM Fluorodeoxyuridine (FUDR), 10 mL:**
0.3693 g	FUDR
10 mL	sterile deionized water
Store at -20 °C.

**Table:** 

**100 mg/mL Ampicillin (Amp), 10 mL:**
1 g	Ampicillin
10 mL	sterile deionized water
Store at -20 °C.

**Table:** 

**50 mg/mL Carbenicillin (Carb), 10 mL:**
500 mg	Carbenicillin
10 mL	sterile deionized water
Store at -20 °C.

**Table:** 

**1 M Isopropyl β-d-Thiogalactopyranoside (IPTG), 10 mL:**
2.38 g	IPTG
10 mL	sterile deionized water
Store at -20 °C.


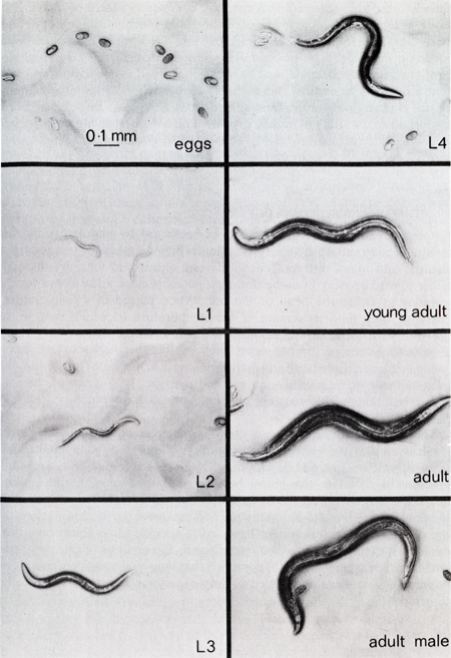
 **Figure 1.** Bright field images of *C. elegans* life stages, including egg, the four larval stages (L1 – L4), and adult. All panels show hermaphrodites except the lower-right, which shows an adult male (Image from Wood (1988)).


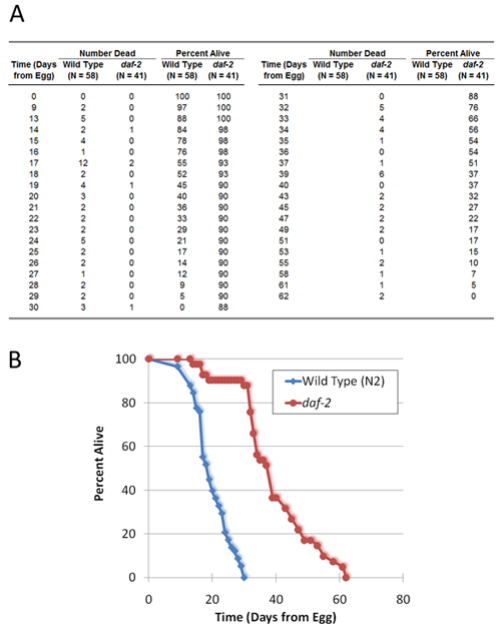
 **Figure 2.** Representative results from a *C. elegans* life span experiment comparing wild type strain N2 to a strain containing a mutation in the *daf-2* gene. (A) A table showing collected data, including number of days since worms were eggs, number of dead worms observed on each day, and the percentage of the original sample remaining alive on each day (as calculated from the daily counts of dead worms). (B) Survival curves corresponding to the life span data provided in (A).


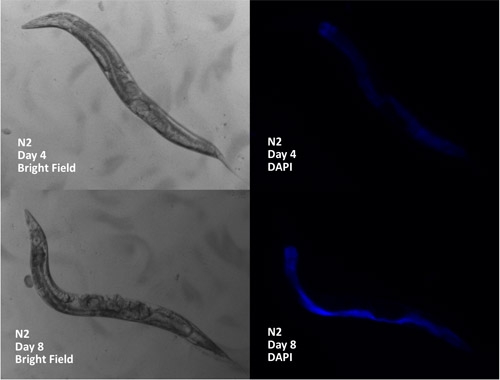
 **Figure 3. **Representative comparison of lipofuscin between young and old adult *C. elegans*. Bright field images are shown on left and DAPI channel fluorescence images on the right. Top panels show a 4 day old worm and bottom panels show an 11 day old worm (as measured from egg).

## Discussion

The genetic control of longevity has been studied extensively in *C. elegans*, largely due to the ease and rapidity with which life span can be determined. The protocol discussed in this article describes a basic framework for obtaining reproducible life span data in *C. elegans* and can also be applied to relatednematode species.^2^ By making some simple alterations these procedures can be adapted to measure life span under a variety of conditions. Here we will discuss several common variations, including live bacteria, RNA interference (RNAi), dietary restriction by bacterial deprivation, and drug-free NGM.

Probably the most common variation from this protocol is the maintenance of worms on live bacteria.  This can be accomplished by making a few minor changes. First, change the procedure for seeding the plates with bacteria (steps 1.7 through 1.13). Instead of growing OP50 cultures to saturation, grow to mid-log phase and pipette 200 µL onto the plates directly from the liquid culture. Allow the bacteria to grow up on the plates over night and omit exposure to UV. Ampicillin should also be excluded from the plates with FUDR. An advantage to using live bacteria is that worms do not have to be transferred as often to new plates, since bacterial food is alive and growing. The disadvantage of live bacterial food is that OP50 is pathogenic to *C. elegans.*^3^ Worms grown on live bacteria have a shorter live span than worms grown on UV-killed bacteria,^3^ which could potentially mask life span phenotypes associated with aging. Median life span on live bacteria is approximately 20 days.

Gene knock down by RNAi is easily accomplished in *C. elegans* by modifying their bacterial food so that it produces double-stranded RNA corresponding to the gene to be knocked down. Two RNAi bacterial libraries are available that cover more than 90% of the open reading frames in the *C. elegans *genome.^4-9^ To utilize RNAi in the context of life span, replace the OP50 bacteria with the RNAi clone of interest and follow the modifications discussed in the previous paragraph for measuring life span on live bacteria. The RNAi libraries use a plasmid based expression system. The plasmid is selected for using a carbenicillin resistance cassette and expression of the double stranded RNA is induced by isopropyl β-d-thiogalactopyranoside (IPTG). Both carbenicillin and IPTG need to be included in the NGM plates. Modify step 1.4 by adding 100 µL 1 M IPTG and 50 µL 50 mg/mL carbenicillin per 100 mL NGM to both types of plates. Ampicillin does not need to be added to either type of plate, since carbenicillin fulfills the same role.

Dietary restriction is the most widely studied intervention for slowing aging across evolutionarily divergent species. In *C. elegans*, maximum life span extension on solid media is observed when the bacterial food source is completely removed during adulthood, a form of dietary restriction termed bacterial deprivation (BD) .^9, 10^ To measure life span in the context of BD, make two modifications to the above protocol. First, prepare a third type of plate. These plates should be identical to the Amp/FUDR plates except lacking the bacterial food source. Second, modify Part 3 to include an additional step between step 3.2 and step 3.3. On the 4th day of adulthood (4 days after transferring the L4 larvae to Amp/FUDR) transfer BD worms to Amp/FUDR plates lacking bacteria. One complication associated with this form of dietary restriction is the worms’ tendency to flee. In the absence of food the worms will not stay near the center of the plate, but will increase their area of exploration in search of food. As a result, a larger fraction of the worms will crawl up the walls of the plate and desiccate. We often see 50% to 70% of the animals flee after being transferred to BD plates. To address this issue, start with three times as many worms in order to have a significant number remaining on the plates post-flight. BD can also be combined with live bacterial food with no additional modifications, or with RNAi with one additional modification. For BD with RNAi, an additional antibiotic must be added to the plates without bacteria to prevent bacteria transferred with the worms from growing and providing an unwanted food source. Two examples are tetracycline and kanamycin, either of which can be added to the FUDR plates without bacterial food during step 1.4.  BD can also be initiated as early as 2 days of adulthood or as late as 14 days of adulthood, with no significant impact on life span.^10 ^

The final modification that we will discuss is measuring life span on NGM plates without additional drugs (e.g. ampicillin or FUDR). This can be accomplished by simply not adding the drugs to the NGM during step 4 and adding one additional step in Part 3. Without FUDR the worms will continue laying eggs and producing larva. During their reproductive phase (approximately the first week of adulthood) all experimental worms will have to be transferred to fresh plates every 2 days to separate them from their offspring.

*C. elegans *are primarily hermaphroditic with rare occurrence of males. Life span is typically measured for hermaphrodites only, but can also be measured for males. There are two challenges associated with working with male *C. elegans*. The first is acquiring a large quantity of male worms, as hermaphrodite self-fertilization produces a very small fraction of male offspring (0.1%).^11^ Once a population containing male worms is attained, male/hermaphrodite mating produces approximately equal numbers of males and hermaphrodites as long as worms are maintained in the presence of food.^12^ Male stocks can be ordered from the *Caenorhabditis Genetics Center *or generated by visually screening for founding males produced from hermaphrodite self-fertilization. The second challenge is male scavenging behavior. In the absence of either food or potential mates (hermaphrodites), male worms enter a searching behavioral mode that involves a wide range of movement.^13^ When maintained on plates this behavior results in a large fraction of the worms fleeing up the plate walls and desiccating. The general method for dealing with this difficulty is simply to start with enough males that a substantial number remain after most have fled.     

Apart from life span, a common age-associated phenotype is lipofuscin accumulation. Lipofuscin is complex cellular waste that cannot be degraded that builds up in cells with age and is used as a biomarker of aging in *C. elegans*.^14, 15 ^Lipofuscin fluoresces and can be easily visualized in *C. elegans* using the DAPI channel of a fluorescence microscope (Figure 3). Lipofuscin accumulation can be visualized in worms being scored for life span directly on the NGM plates, allowing collection of a useful secondary phenotype in parallel with life span.

In addition to life span, the protocol described in this article can also be used to score phenotypic progression of age-associated paralysis in *C. elegans *models of proteotoxicity disease.^16^ When a worm becomes paralyzed it becomes unable to crawl across the plate, but can still move its head. A worm is scored as paralyzed if it fails to move relative to the NGM in response to plate tapping or prodding with a platinum transfer pick, but does move its head. Worms that die typically retain the paralysis score (paralyzed or not paralyzed) that they were given during the most recent live observation. Importantly, even wild type *C. elegans* become paralyzed with advanced age. For this reason paralysis is typically not scored for worms older than approximately 20 days, as beyond this point it becomes difficult to distinguish between paralysis caused by normal aging and paralysis caused by progression of the proteotoxicity disease.

## References

[B0] Wood WB, Wood WB (1988). The Nematode Caenorhabditis elegans.

[B1] Sutphin GL, Kaeberlein M (2008). Dietary restriction by bacterial deprivation increases life span in wild-derived nematodes. Exp Gerontol.

[B2] Garigan D (2002). Genetic analysis of tissue aging in Caenorhabditis elegans: a role for heat-shock factor and bacterial proliferation. Genetics.

[B3] Chen D, Pan KZ, Palter JE, Kapahi P (2007). Longevity determined by developmental arrest genes in Caenorhabditis elegans. Aging Cell.

[B4] Curran SP, Ruvkun G (2007). Lifespan regulation by evolutionarily conserved genes essential for viability. PLoS Genet.

[B5] Dillin A (2002). Rates of behavior and aging specified by mitochondrial function during development. Science.

[B6] Hamilton B (2005). A systematic RNAi screen for longevity genes in C. elegans. Genes Dev.

[B7] Hansen M, Hsu AL, Dillin A, Kenyon C (2005). New genes tied to endocrine, metabolic, and dietary regulation of lifespan from a Caenorhabditis elegans genomic RNAi screen. PLoS Genet.

[B8] Lee SS (2003). A systematic RNAi screen identifies a critical role for mitochondria in C. elegans longevity. Nat Genet.

[B9] Smith ED (2008). Age- and calorie-independent life span extension from dietary restriction by bacterial deprivation in Caenorhabditis elegans. BMC Dev Biol.

[B10] Hodgkin J, Horvitz HR, Brenner S (1979). Nondisjunction Mutants of the Nematode CAENORHABDITIS ELEGANS. Genetics.

[B11] Emmons SW, Sternberg PW, Riddle DL, Blumenthal T, Meyer BJ, Priess JR (1997). C. ELEGANS II.

[B12] Lipton J, Kleemann G, Ghosh R, Lints R, Emmons SW (2004). Mate searching in Caenorhabditis elegans: a genetic model for sex drive in a simple invertebrate. J Neurosci.

[B13] Davis BO, Anderson GL, Dusenbery DB (1982). Total luminescence spectroscopy of fluorescence changes during aging in Caenorhabditis elegans. Biochemistry.

[B14] Klass MR (1977). Aging in the nematode Caenorhabditis elegans: major biological and environmental factors influencing life span. Mech Ageing Dev.

[B15] Steinkraus KA (2008). Dietary restriction suppresses proteotoxicity and enhances longevity by an hsf-1-dependent mechanism in Caenorhabditis elegans. Aging Cell.

